# A Hybrid Ensemble Approach for Identifying Robust Differentially Methylated Loci in Pan-Cancers

**DOI:** 10.3389/fgene.2019.00774

**Published:** 2019-09-05

**Authors:** Qi Tian, Jianxiao Zou, Yuan Fang, Zhongli Yu, Jianxiong Tang, Ying Song, Shicai Fan

**Affiliations:** ^1^School of Automation Engineering, University of Electronic Science and Technology of China; ^2^Center for Informational Biology, University of Electronic Science and Technology of China, Chengdu, China

**Keywords:** DNA methylation, differentially methylated loci, ensemble feature selection, robustness, pan-cancers

## Abstract

DNA methylation is a widely investigated epigenetic mark that plays a vital role in tumorigenesis. Advancements in high-throughput assays, such as the Infinium 450K platform, provide genome-scale DNA methylation landscapes in single-CpG locus resolution, and the identification of differentially methylated loci has become an insightful approach to deepen our understanding of cancers. However, the situation with extremely unbalanced numbers of samples and loci (approximately 1:1,000) makes it rather difficult to explore differential methylation between the sick and the normal. In this article, a hybrid approach based on ensemble feature selection for identifying differentially methylated loci (HyDML) was proposed by incorporating instance perturbation and multiple function models. Experiments on data from The Cancer Genome Atlas showed that HyDML not only achieved effective DML identification, but also outperformed the single-feature selection approach in terms of classification performance and the robustness of feature selection. The intensive analysis of the DML indicated that different types of cancers have mutual patterns, and the stable DML sharing in pan-cancers is of the great potential to be biomarkers, which may strengthen the confidence of domain experts to implement biological validations.

## Introduction

DNA methylation is one of the essential epigenetic mechanisms, which plays a vital role in normal development and is closely correlated with the cell growth, differentiation, and transformation in eukaryotes (Robertson, 2005; Suzuki and Bird, 2008; Laird, 2010; Jones, 2012).Failure of proper maintenance of epigenetic marks, like abnormal DNA methylation, may result in inappropriate activation or inhibition of various signaling pathways, leading to diseased states, even cancers (Esteller, 2007; Hanahan and Weinberg, 2011; Dawson and Kouzarides, 2012; Aran and Hellman, 2013; Tolstorukov et al., 2013). For example, aberrant promoter hypermethylation that is associated with inappropriate gene silencing affects virtually every step in tumor progression (Jones and Baylin, 2002). So, the investigation of differential methylation, which displays the inherent difference between normal and tumor samples, could help us deepen our perception of oncogenesis and may assist in the early diagnosis of cancers (Tost, 2007; Deng et al., 2010).

High-throughput bisulfite sequencing provides a new stage for researchers to analyze methylation variability at single-base resolution, and the identification of differentially methylated loci (DML) has become an insightful attempt for detection of tumor markers (Cokus et al., 2008; Down et al., 2008). In the early stage, obtaining methylation data is based on bisulfite sequence technique (BS-seq), and Lister et al. (2009) first use Fisher exact test to select differential methylation sites. Then, more R packages have been developed for identifying DML with this kind of data. BiSeq (Hebestreit et al., 2013) and DSS (Feng et al., 2014) concentrate on identifying DML through Wald tests, whereas MethylSig (Park et al., 2014) applies likelihood ratio tests for DML identification. Infinium HumanMethylation450 BeadChip is now widely used in methylation analysis for its advantages of lower cost and easier experimental protocol compared with BS-seq, like WGBS, and is suggested to be suitable for large-scale studies (Dedeurwaerder et al., 2011). For example, IMA achieves detection of site-level differential methylation using Wilcoxon rank-sum tests with HM450 data (Wang et al., 2012). Compared with IMA, based on the analysis of covariance, FastDMA performs better in identifying DML with higher computational efficiency (Wu et al., 2013). RnBeads provides a comprehensive pipeline for analysis and interpretation of DNA methylation with *t* statistics analysis based on linear model and empirical Bayes (Assenov et al., 2014). We consider that the identification of DML is to search for loci that can significantly distinguish between the normal and the sick, and therefore the essence of this problem can be regarded as applying feature selection to the identification of DML. Additionally, compared with the methods mentioned above, feature selection approaches can take the feature redundance and irrelevance into account, and this could be a benefit for selecting more significant DML.

However, considering that the HM450 data have a small number of samples but high dimensional features (approximately 1:1,000), the results from general feature selection methods for identifying DML will have poor robustness (Kim, 2009). The robustness (reproducibility or stability) of selected loci is extremely important for identifying DML, as domain experts tend to do subsequent analysis and validations with stable results. While feature selection has been considered a de facto standard in microarray data mining (Bolon-Canedo et al., 2014), how to identify robust DML with feature selection has received little attention. Recent advancements in ensemble feature selection provide a promising approach to solve the robustness problem in large-scale biological data (Saeys et al., 2008; Abeel et al., 2010; Liu et al., 2010; Yang et al., 2010; Haury et al., 2011; Yang et al., 2011; Yu et al., 2012). The rationale for this idea is combining single, less stable feature selectors to yield a more robust one, which is the same as ensemble learning: in a first step, a number of different feature selectors are used, and in a final phase, the output of these separate selectors is aggregated and returned as the final (ensemble) result. Specifically, there are two major means to achieve ensemble feature selection; one of them is data diversity (instance perturbation), which uses the same feature selection method on different data subsets from multiple sampling on the original data set, and the other is function diversity, which implements different feature selection methods on the original data set (Saeys et al., 2008; Yang et al., 2010; Awada et al., 2012; Yu et al., 2012).

In this article, we aggregate data diversity and function diversity to propose a hybrid ensemble approach for identification of DML (HyDML). Under the framework of ensemble feature selection, this newly proposed method not only can realize the effective identification of DML, but also can accommodate for the robustness of the results. Additionally, taking advantage of the large-scale Infinium 450K methylation data produced by The Cancer Genome Atlas (TCGA) project, we performed intensive analysis to look further into interrelationships between differential methylation and cancers and found that different cancers have common patterns, and robust DML sharing in pan-cancers is of the great potential to be biomarkers.

## Materials and Methods

### Cancers and Samples

For feeding the algorithm and analysis, in total 13 cancers are selected with both normal and tumor samples. Specifically, these cancers are bladder urothelial carcinoma (BLCA), breast invasive carcinoma (BRCA), colon adenocarcinoma (COAD), esophageal carcinoma (ESCA), head and neck squamous cell carcinoma (HNSC), kidney renal clear cell carcinoma (KIRC), kidney renal papillary cell carcinoma (KIRP), liver hepatocellular carcinoma (LIHC), lung adenocarcinoma (LUAD), lung squamous cell carcinoma (LUSC), prostate adenocarcinoma (PRAD), thyroid carcinoma (THCA), and uterine corpus endometrial carcinoma (UCEC). In all, there are 6,189 samples including 699 normal samples and 5491 tumor samples (**Table S1**).

### DNA Methylation Data and Preprocess

We downloaded the DNA methylation data from TCGA data portal (https://tcga-data.nci.nih.gov/tcga/) for our selected samples. The methylation data are generated by Illumina Infinium HumanMethylation450k BeadChip technique. The Illumina Infinium assay utilizes a pair of probes for each CpG site, one probe for the methylated allele and the other for the unmethylated version. The methylation level is then estimated, based on the measured intensities of this pair of probes, as the ratio of methylated signal to the sum of methylated and unmethylated signal, which ranges from 0 (absent methylation) to 1 (completely methylated). To assess the ability of the selected DML to distinguish between the two types of samples (tumor and normal), we retrieved three independent test sets from the NCBI database. The three data sets are also obtained by HM 450 technique, including samples of breast (GSE52635), liver (GSE54503), and lung (GSE66836) cancer, as well as corresponding normal tissue data records (**Table S1**). For each type of cancer, the original methylation data record the methylation level at more than 450,000 loci. A series of preprocessing is required before implementing the selection of DML, which can reduce the computational complexity as well as improve the accuracy of the final results. The preprocessing steps for the methylation data are as follows: i) The 450k methylation chip uses two different types of probes (type I and type II) when measuring the locus methylation and results in two different types of data distribution. We use the SWAN algorithm to eliminate the abiotic variation caused by the measurement of the two probes while preserving the biological differences of the samples (Maksimovic et al., 2012). ii) Eliminate batch effects caused by system bulk effects or abiotic differences using empirical Bayesian (EB) methods (Johnson et al., 2007). iii) Filter out some of the minimal variance loci to avoid dimensionality disasters and remove significantly unrelated redundant loci. After completing all of the preprocessing steps, approximately 350,000 feature sites are obtained for each cancer for subsequent feature selection. Considering polymorphisms (single-nucleotide polymorphisms), we chose to mark these sites in the results, and users can decide the stringency of probe filtering appropriate for their analysis.

### Hybrid Ensemble Approach for Identification of DML

**Figure 1:**
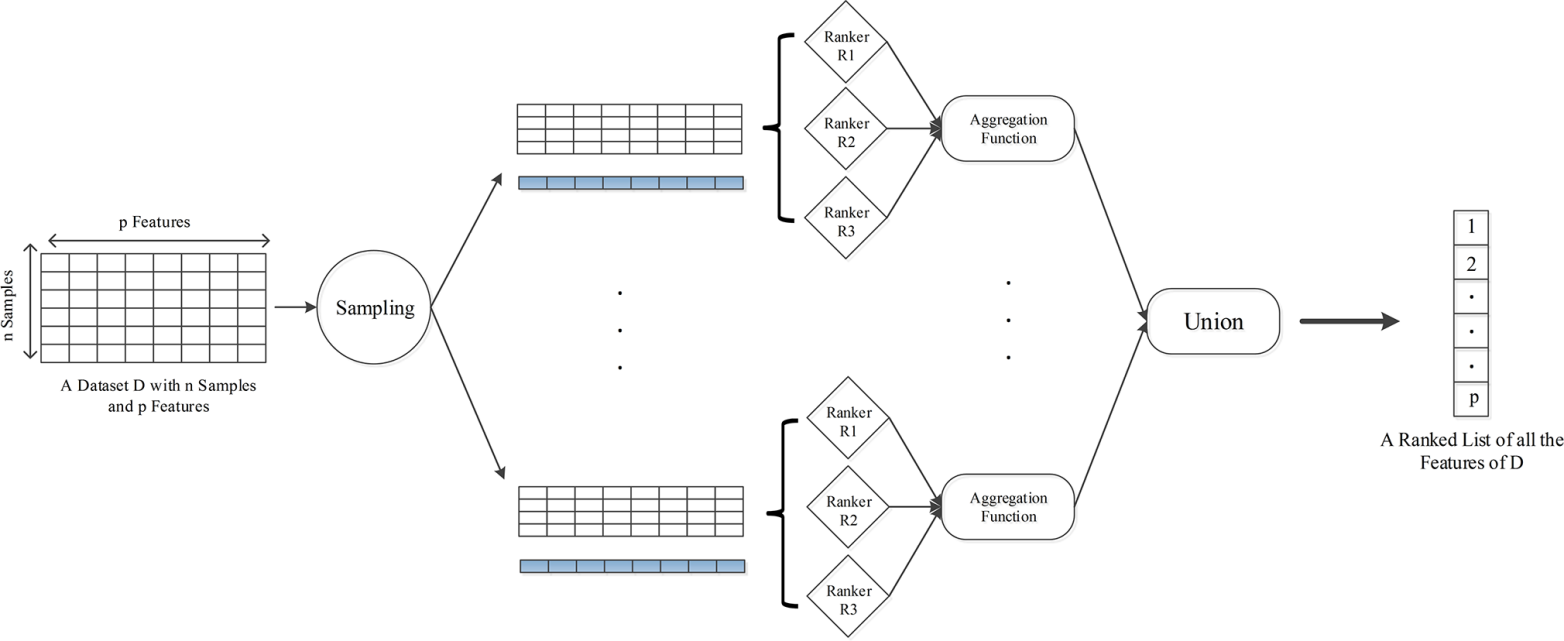
The framework of HyDML for identifying differentially methylated loci using hybrid ensemble feature selection approach.

First, in order to obtain a diverse set of feature selectors, we perform multiple samplings on training samples to generate data subsets. To this end, we make use of resampling and cross-validation, integrating classifier training into the ensemble feature selection framework for selecting loci that are informative for classifying tumor and normal samples. In each sampling, the whole data set is divided into 10 pieces with the same number of samples, and each of them can be regarded as a test subset to validate subsequent classification performance, while the rest automatically becomes a training set for feature selection and classifier training (constructed with support vector machine) (Cortes and Vapnik, 1995). The instance level perturbation here can bring in the stability for feature selection after aggregating the result of each data subset, because the stable features are more likely to appear in different training subsets when the sample changes slightly. Then, generating functional diversity is achieved by using multiple feature selection methods on the same training set. With consideration of high dimensionality and small sample size of the 450k methylation data, embedded feature selection methods could be a practical choice for the appropriate computation complexity. Thus, we choose R packages “glmnet,” “MDFS” and “rmcfs” as the basic feature selection approaches (Friedman et al., 2010; Draminski and Koronacki, 2018; Piliszek et al., 2018). Taking the advantages of combing L1 and L2 regularization (elastic net), glmnet can achieve variable extraction for the microarray data with high dimension but small number of samples. Combining linear model with elastic net for feature selection, the optimization function is as follows:

$$\arg\underset{w}{min}\left\{\overset{m}{\underset{i=1}{\sum }}{\left(y_{i}-w^{T}x_{i}\right)}^{2}+\lambda \left[\alpha \overset{p}{\underset{j=1}{\sum }}\left|w_{j}\right|+\left(1-\alpha \right)\overset{p}{\underset{j=1}{\sum }}w_{j}^{2}\right]\right\}$$

where w represents the feature weight coefficient, *m* represents the number of samples, and *p* represents the total number of features in the data set. λ is used to balance the empirical risk and model complexity, whereas α is used to balance the regularization of L1 and L2. In MDFS, we apply feature selection with max information gain criterion, which measures the worth of a feature by computing the information gain values with respect to the class. For rmcfs, it relies on a Monte Carlo approach to select informative features and is capable of incorporating interdependencies between features. The three basic feature selection algorithms can be well adapted to the high-dimensional and small-sample characteristics of 450k methylation data, and the whole calculation amount is moderate, while classification performance can be guaranteed. For each data subset, aggregating the results of multiple feature selection methods could further enhance the stability. More formally, consider an ensemble feature selector *E* = {*F*_1_, *F*_2_,… ,*F* *_s_*} and each *F* *_i_* provides a feature ranking $f_{i}=\left(f_{i}^{1},f_{i}^{2},\ldots ,f_{i}^{N}\right)$, *f* *_i_* denotes the feature weight of each *F* *_i_* and *N* represents the nth feature. Hence, a general aggregation formulation for the ensemble ranking **f**, obtained by weighted summing the ranks over all **f** *_i_*, is as follows:

$$f=\overset{s}{\underset{i=1}{\sum }}{acc}_{i}^{*}f_{i}$$

where *acc* *_i_* donates the accuracy of the corresponding test set on the classifier trained by feature selector *F* *_i_*, and **f** also can be regarded as the aggregation ranking for the ensemble feature selector. Here, s = 3, which represents the three basic feature selection methods, and we can get the preliminary DML at this level of aggregation. Then, taking the union set of obtained loci subsets is the second level of aggregation, and the corresponding formula representation is as follows:

$$f=\overset{s}{\underset{i=1}{\sum }}f_{i}$$

where *s* donates the number of data subsets, and **f** *_i_* is the feature ranking of corresponding data subset. In this way, one aggregated ranking of all the features for each sampling can be yielded. We perform 10 iterations for generating more data subsets to further improve the stability of selected loci, and with the idea of bagging, the final DML set consisted of loci that appear more than five times in 10 iterations. The overall algorithm framework for one sampling is shown in **Figure 1**, and pseudo code flow is as follows:

**Table:** Algorithm: Hydml

**Require:** methylation data **D**
**Ensure:** Divide data set D into {***D***_1_, ***D***_2_,…, ***D*** ***_k_***,…***D***_10_} for 10-fold cross-validation;
1: begin
2: **for** k = 1 to 10 **do**. The data subset ***D*** ***_k_*** is used as a **test set**, while other data subsets are used as a **training set** to produce DML with **multiple feature selection methods**; calculate $f_{i}^{k}f_{k}$ for each feature in ***D*** ***_k_*** with ***acc*** ***_i_***(***i*** = 1, 2, 3); filter out loci with the **f** ***_k_*** < 0.01; **end for**;
3: Take **union** set of {***f***_1_, ***f***_2_,,, ***f***_10_} to obtain **F**_1_
4: **for** *t* = 1 to 10 **do**, step 2 and step 3; **end for**;
5: Aggregate ***F***_1_ ∼ ***F***_10_ with bagging which filters out loci which appears less than five times; record as ***F***
6: **return** ***F***;
7: **End**

## Performance Evaluation and Comparison

### Stability Measure

To measure the effect of our hybrid ensemble technique on the feature selection results, following Saeys et al. (2008), we take a similarity-based approach where feature stability is measured by comparing the signatures from the k feature selectors. The more similar all signatures are, the higher the stability measure will be. The overall stability can be defined as the average over all pairwise similarity comparisons between different signatures:

$$S_{tot}=\frac{2\sum_{i=1}^{k}\sum_{j=i+1}^{k}S\left(f_{i},f_{j}\right)}{k\left(k-1\right)}$$

where *f* *_i_* represents the signature obtained by the selection method on subsampling *i*(1 ≤ *i* ≤ *k*); *k* is the number of data subsets; *S*(*f* *_i_*, *f* *_j_*) is a similarity measure for feature subsets, which denotes the stability of *f* *_i_* and *f* *_j_*. Here, we use Jaccard index (Saeys et al., 2008) as *S*(*f* *_i_*, *f* *_j_*):

$$S\left(f_{i},f_{j}\right)=\frac{\left|f_{i}\cap^{\text{​}}f_{j}\right|}{\left|f_{i}\cup^{\text{​}}f_{j}\right|}=\frac{\sum_{l}I\left(f_{i}^{l}=f_{j}^{l}=1\right)}{\sum_{l}I(f_{i}^{l}+f_{j}^{l}>0)}$$

where the indicator function I(.) returns 1 if its argument is true, and zero otherwise. In the sequel, the overall stability *S* *_tot_* is simply denoted by *S*(*f* *_i_*, *f* *_j_*).

### Classification Performance Measure

To evaluate the classification performance and perform comparisons, we use several characteristics of classification performance all derived from the confusion matrix. These characteristics are TP, TN, FP, and FP, which denote true-negatives, true-positives, false-negatives, and false-positives, respectively. All the performance metrics are calculated by these characteristics, including TPR (true-positive rate), FPR (false-negative rate), ACC (classification accuracy), Precision, Recall, and F1 score. We also include the area under the receive operating characteristic curve, which is defined by a function of sensitivity and specificity, further abbreviated as AUC.

## Results

### Characteristics of Differentially Methylated Loci in 13 Cancers

**Figure 2:**
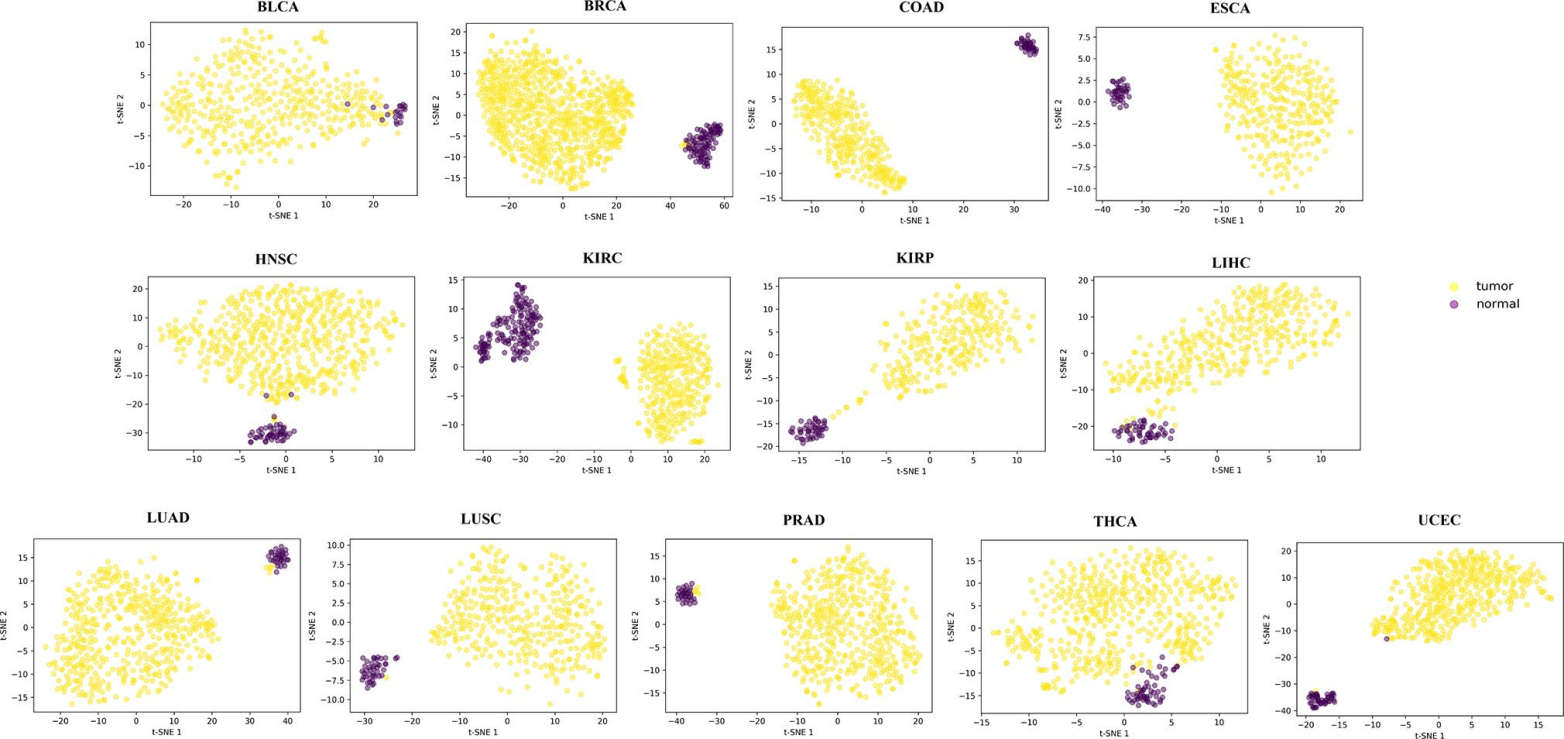
The clustering results by t-SNE using the obtained differential methylation sites for each cancer.

For each of the 13 cancers, we finally obtained a set of DML, which varies from 5,700 in COAD to 14,516 in THCA (**Table S2**). Through t-SNE clustering (van der Maaten and Hinton, 2008), we found that these differential methylation sites were able to distinguish the difference between the normal and the sick, especially in COAD, ESCA, and KIRC (**Figure 2**). While very few samples were misclassified, it was probably due to the information compression since the original feature dimension is reduced by thousands of times during the t-SNE clustering process.

We first explored the distribution of DML in 22 pairs of autosomes for each cancer, which could help us to find out which chromosome gets potential extensive genetic variation when cancer occurs. To this end, we calculated the distribution density of the DML on each autosome, using ratio of the number of DML to the number of CpG sites determined by the 450K chip (**Figure S1A**). We can see from the results that chromosome 20 was enriched with more sites, whereas the DML were less distributed on chromosome 1, 9, oppositely. Combining the functional regions of genes on the chromosome, we further analyzed the distribution of DML in the promoter region (regions from 2,000 bps upstream to the transcription start site), gene body (excluding promoter region), and intergenic region for each cancer. Most of DML were located in nonpromoter regions (gene body and intergenic region; **Figure S1B**). However, considering that the promoter region occupied only a small part of the genome, the number of DML accounted for more than 20%, indicating that the abnormal methylation of this short functional region had an important impact on the tumorigenesis (Jones and Baylin, 2002; Baylin and Ohm, 2006). Most DML were distributed on CpG islands (**Figure S1C**), which has been reported that aberrant methylation of CpG islands was related to transcriptional gene silencing or activation of multiple oncogenes (Costello et al., 2000; Chan et al., 2002; Klutstein et al., 2016; Soozangar et al., 2018).

We also observed that biologically similar cancers shared more mutual DML through hierarchical clustering using similarity metric based on Jaccard index (**Figure S2**). Specifically, smoking- and drug addiction-related cancers, like LUSC and HNSC, were clustered together (Brennan et al., 1995; Johnson et al., 2005; Campbell et al., 2016). KIRC and KIRP were both due to renal lesion. High-risk cancers that were predisposed to women, such as BRCA and UCEC, shared more DML and were clustered together.

### Robust Feature Selection Improves the Classification Performance

**Figure 3:**
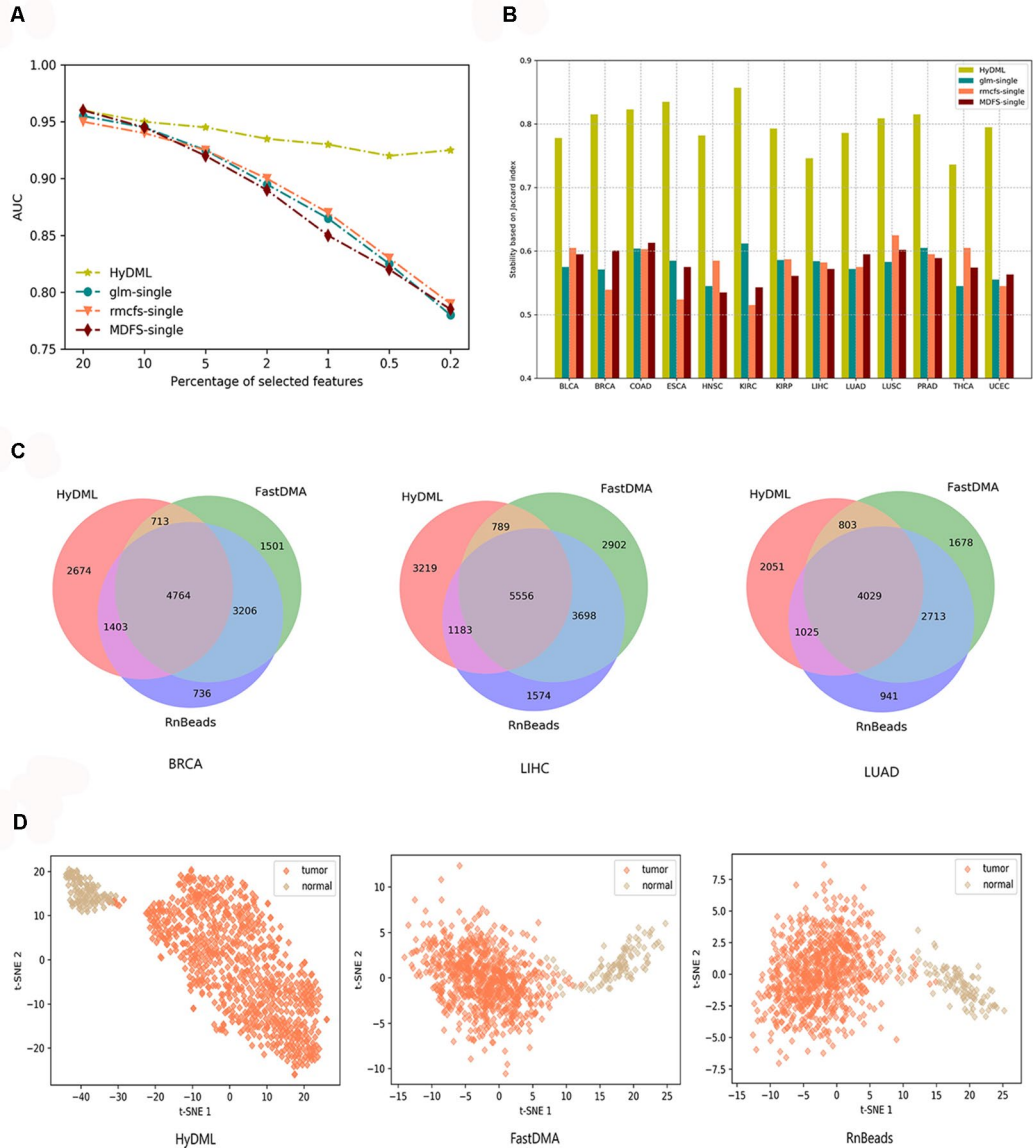
Thorough classification performance and the robustness measure to compare different models in identifying differential methylation sites. **(A)** Classification performance of HyDML and its corresponding submethods as the selected features (loci) gradually reduce in PRAD. **(B)** Comparison of robustness measure using Jaccard index in 13 cancers for HyDML and its corresponding submethods. **(C)** Relationship of differential methylation sites obtained by HyDML/FastDMA/RnBeads in BRCA; LIHC and LUAD (corresponding to the three independent test sets) using a Venn picture. **(D)** T-SNE clustering results in BRCA using the unique differential methylation sites selected by each method.

First, we compared our newly proposed method to its baseline methods, glmnet, rmcfs, and MDFS when the number of loci gradually decreased. This could help us analyze the robustness of the results from different feature selection methods as the features reduced, or if a feature selection method could identify more robust features, the decrement of features would not have a significant impact on the results. Here, for the three baseline methods, the feature sets were produced by a default configuration. Using the comprehensive classification metric, AUC, **Figure 3A** displays the trend of AUC change as the feature number reduced on PRAD data set. It can be observed that our ensemble approach clearly improved upon the baselines in terms of classification performance as the loci decreased. We also implemented the comparison on data of the other 12 cancers, and the results showed that the hybrid ensemble framework was superior to single-feature selection methods, thus demonstrating that the ensemble methods were better capable of eliminating noisy and irrelevant dimensions (**Figure S3**). We also compared the stability or robustness measure *S* *_tot_* (based on Jaccard Index, see *Materials and Methods*), and the results in all 13 cancers showed the hybrid ensemble approach (HyDML) performed better than single-feature selection methods, which could be a benefit in performing subsequent analysis with the selected differential methylation sites (**Figure 3B**).

Moreover, three independent test sets from the NCBI database (BRCA: GSE52635; LIHC: GSE54503; LUAD: GSE66836) were used to compare HyDML with classical DML identification methods, including FastDMA and RnBeads, for analyzing the differences between the ensemble feature selection approach and the statistical test method. Using the original DML previously selected from the three cancers as training sets, we constructed a classification model based on SVM and performed the verification with the test sets. The results showed that DML selected by HyDML performed better than FastDMA and RnBeads (**Table 1**). Compared with the two classical DML finding approaches, the selected feature from HyDML showed better generalization ability in distinguishing the normal and tumor samples. Then, we analyzed the loci selected by the three methods to verify whether the loci were distinct from each other. Experiments on data of the three cancers showed that most DML were identical for the three methods, whereas FastDMA and RnBeads shared more mutual DML (**Figure 3C**). To capture the key differences of the three methods, we further studied the DML, which were uniquely selected by the corresponding method (the loci selected by one of the methods and not selected by the other two methods), through t-SNE clustering, and the results of BRCA showed that the uniquely selected DML from HyDML were more able to describe the difference between the normal and the sick (**Figure 3D**). The clustering results of the other two cancers can be obtained in **Figure S4**, and HyDML not surprisingly displayed better performance in classifying normal and tumor samples. This indicated that the differential methylation sites obtained by the hybrid ensemble approach were more likely to be reliable in biological validations. One evident reason for this was that HyDML takes the robustness of selected loci into account, and this could be rewarding to produce better DML in terms of analyzing the difference between the normal and the sick.

**Table 1:** Classification performance comparison on three independent test sets.

GSE52635							
	TPR	FPR	ACC	AUC	Precision	Recall	F1
FastDMA	0.958	0.083	0.938	0.924	0.921	0.958	0.939
RnBeads	0.938	0.042	0.948	0.935	0.957	0.938	0.947
HyDML	**0.979**	**0.042**	**0.969**	**0.968**	**0.959**	**0.979**	**0.969**
GSE54503							
	TPR	FPR	ACC	AUC	Precision	Recall	F1
FastDMA	0.909	0.1667	0.8712	0.897	0.779	0.909	0.839
RnBeads	0.955	0.1515	0.9016	0.923	0.863	0.955	0.906
HyDML	**0.969**	**0.091**	**0.9408**	**0.962**	**0.914**	**0.969**	**0.941**
GSE66836							
	TPR	FPR	ACC	AUC	Precision	Recall	F1
FastDMA	0.909	0.316	0.886	0.876	0.961	0.909	0.934
RnBeads	0.915	0.263	0.896	0.893	0.968	0.915	0.940
HyDML	**0.951**	**0.158**	**0.94**	**0.943**	**0.981**	**0.951**	**0.966**

In bold font: best performance.

### Pan-Cancer–Related DML Provide a Landscape of Commonality in Different Cancers

**Figure 4:**
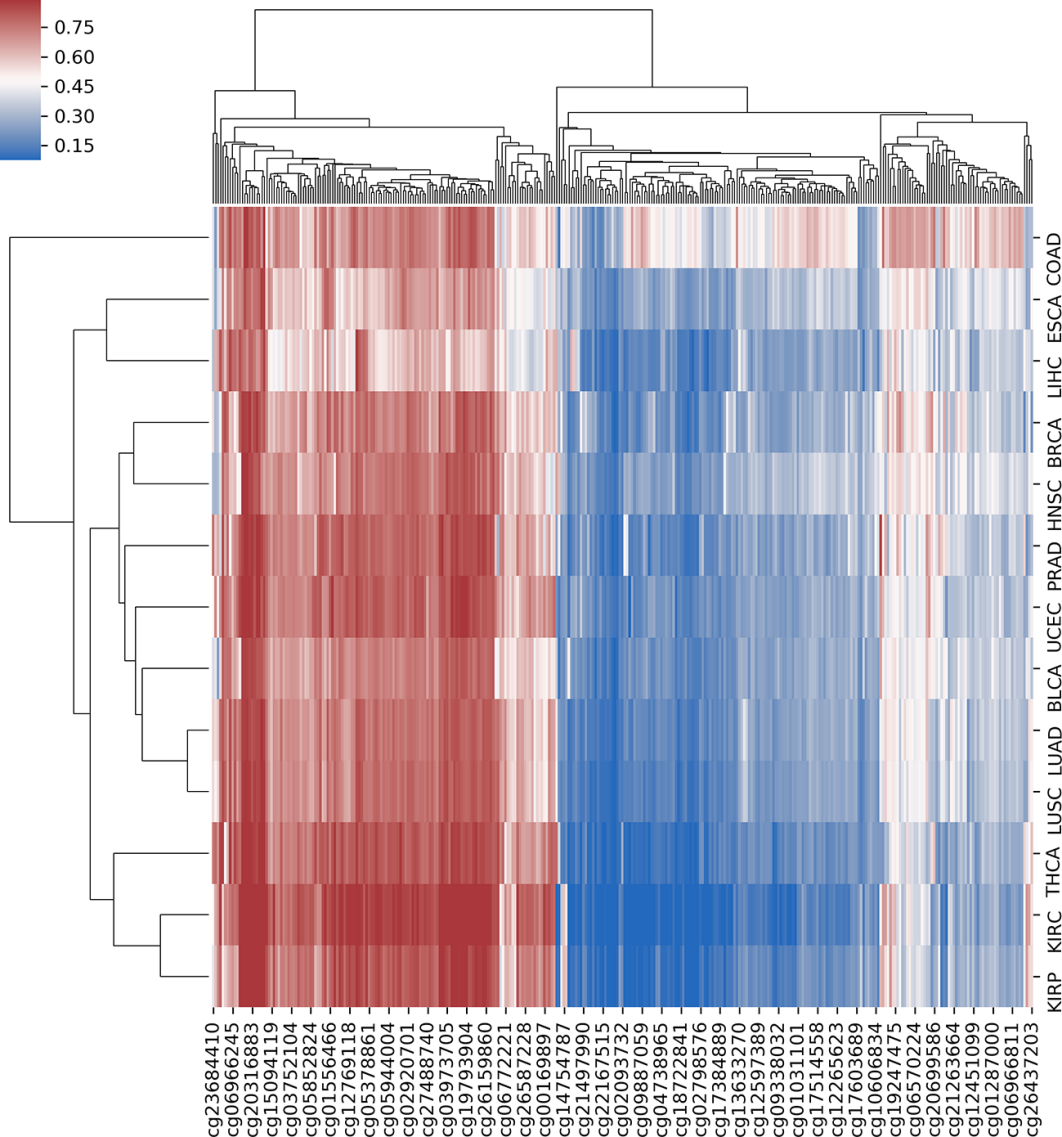
The hierarchical clustering with heat map using all predefined pan-cancer differential methylation sites in 13 cancers.

In order to further analyze the association between DNA methylation and cancer, we investigated the differential methylation sites that occurred in multiple cancers, which could help us reveal the pan-cancer–associated methylation patterns. First, we defined a selected site as a pan-cancer differentially methylated locus (pDML) if it occurred no less than 10 times in 13 cancers. We in total obtained 338 pDML, in which some of them presented as hypermethylated, whereas the others presented obvious hypomethylation, expressed by median value in normal and tumor samples (**Table S3**). By combining the methylation expression levels of pDML in tumor samples, different cancers reflected similarities in methylation variation (**Figure 4**). For example, LUAD and LUSC were clustered together as a result of carcinogenesis of lung tissues, and kidney disease–related cancer, such as KIRC and KIRP, were also shown to be similar in terms of pDML. This verified the methylation specificity expression caused by the differentiation of tissues, and even when the tissues were cancerous, there was a certain degree of difference in methylation variability between tissues, or the cancer subtypes of the same tissue had more similar methylation patterns.

**Figure 5:**
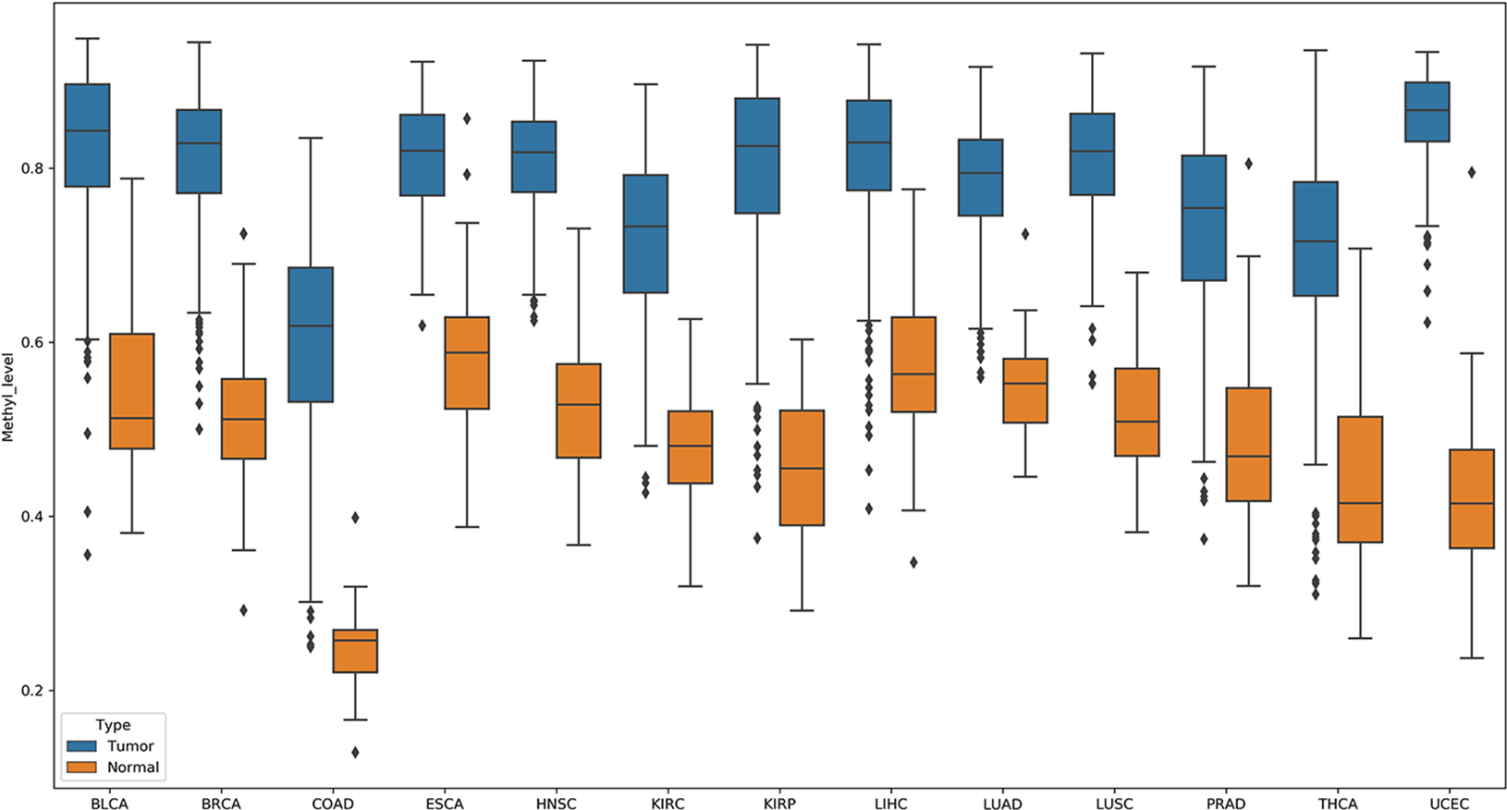
Probe cg02829688 showed significant hypermethylation (the methylation level of loci in tumor samples were higher than those in normal samples) in all 13 cancers.

In these pDML, we also found that, one probe, cg02829688, was significantly hypermethylated (the methylation level of loci in tumor samples was higher than that in normal samples) in all 13 cancers (**Figure 5**). Through the annotation files, we found that it was located at chr1:119527008 in a CpG island and belonged to a differentially methylated region (experimentally determined). Moreover, the corresponding upstream and downstream regions were located in a target gene, *TBX15*. It has been demonstrated that *TBX15* plays a vital role in multiple cancers, such as non–small cell lung cancer (Carvalho et al., 2013), thyroid cancer (Arribas et al., 2015), and ovarian carcinoma (Gozzi et al., 2016), and especially has been proved to be a methylation marker of prostate cancer (Kron et al., 2012). Moreover, Chelbi et al. (2011) identified a region located in the distal promoter of the *TBX15* that was differentially methylated and suggested that *TBX15* might be involved in the pathophysiology of placental diseases.

**Figure 6:**
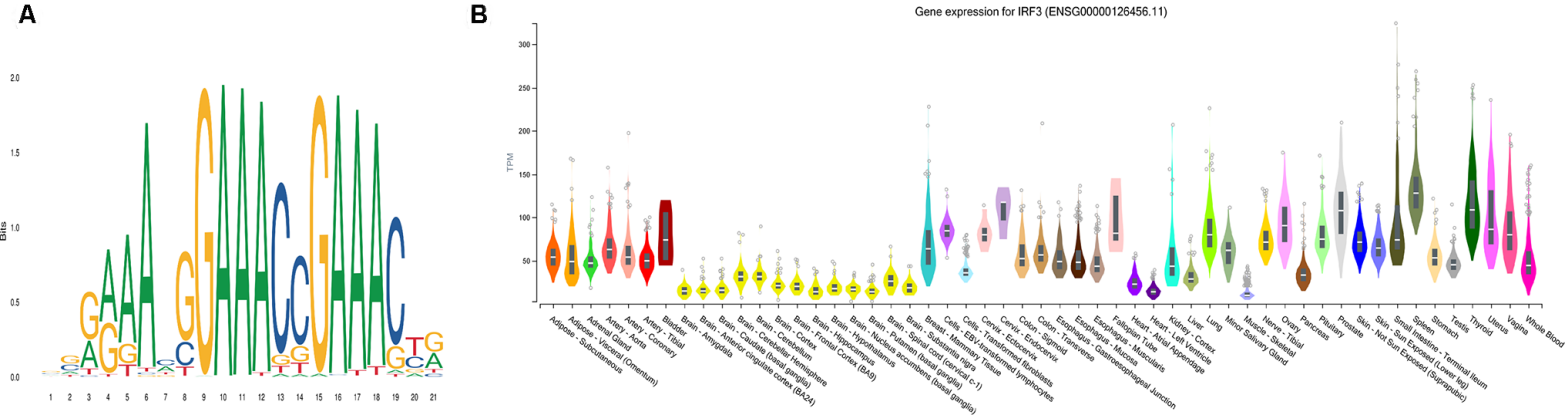
The most significantly enriched motif, IRF3. **(A)** The motif logo of IRF3. **(B)** The gene expression for IRF3 in multiple tissues.

Using AME (McLeay and Bailey, 2010), the motif enrichment tool of MEME Suite, we detected sequence motifs that were enriched in the background sequences generated from the pDML, which were located in promoter regions and identified 84 motifs (**Table S4**). The motif of IRF3 was the most significantly enriched one (*P* = 5.55e-21) (**Figure 6A**), and the gene expression for IRF3 has been experimentally determined in multiple tissues (**Figure 6B**). IRF3 as a transcription factor has been reported as a regulator in type I interferon genes playing a vital role in mammalian response to pathogens and considered to be implicated in various biological pathological conditions, including cancer (Wang et al., 2017; Andrilenas et al., 2018). Baylin et al. (2006) also demonstrated that DNA methyltransferase inhibitors triggered viral defense and induced IRF3 to translocate to the nucleus and activated transcription of IFNβ1 to influence immune signaling in cancers (Chiappinelli et al., 2015).

**Figure 7:**
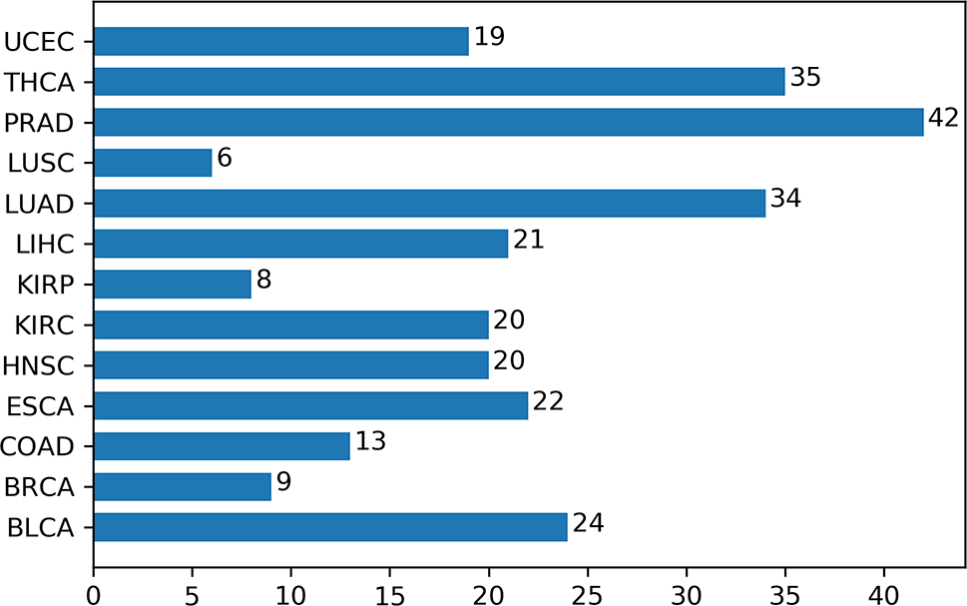
The number of metabolic pathways for DML-associated genes in each cancer.

Additionally, we had a deeper insight into the relationship between methylation and cancers through analyzing the corresponding biological pathways. Using the KEGG pathway database (Kanehisa and Goto, 2000), **Figure 7** showed the number of metabolic pathways for DML-associated genes in each cancer (*P* < 0.05). Then, we summarized the pathways that occurred in at least seven cancers and denoted as pan-cancer methylation-related pathways (PMPs) and obtained in total 11 PMPs, where 10 of them have been reported to be associated with cancers (**Table 2**). The only one PMP, neuroactive ligand-receptor interaction, has not been proven to be directly or indirectly associated with cancers, but further research is needed for deeper exploration.

**Table 2:** PMPs and their corresponding relations to cancer.

Pan-cancer methylation related pathways	Related to cancer?
Antigen processing and presentation (Chapman et al., 2013)	Yes
Allograft rejection (Leone et al., 2013)	Yes
cAMP signaling pathway (Fajardo et al., 2014)	Yes
Cell adhesion molecules (Okegawa et al., 2004)	Yes
Graft-versus-host disease (Curtis et al., 2005)	Yes
MicroRNAs in cancer (Wiemer, 2007)	Yes
Nicotine addiction (Benowitz, 2009)	Yes
Rap1 signaling pathway (Zhang et al., 2017)	Yes
Type II diabetes mellitus (Shlomai et al., 2016)	Yes
Autoimmune thyroid disease (Turken et al., 2003)	Yes

## Discussion

Identifying DML is a promising approach to reveal the inherent intricacy between aberrant DNA methylation and tumorigenesis, and recent studies have paid more attention to this essential epigenetic mechanism. Taking advantage of the large-scale DNA methylation data produced by TCGA, we investigated the differential methylation in 13 cancers with a newly proposed approach under hybrid ensemble feature selection framework. Compared with single-feature selection methods in identifying DML, HyDML could achieve identifying more robust loci, and the improvement of reproducibility of feature selection algorithm’s results can enhance the confidence of researchers in experimental verification, especially in finding biomarkers. Compared with classical DML identification methods based on traditional statistic theory (such as FastDMA and RnBeads), feature selection–based approaches could select more informative loci that are closely related to the difference between the normal and the sick, as well as eliminating noisy and irrelevant loci, especially when dealing with microarray data of sparse samples and high-dimensional features. By t-SNE clustering, the results showed that the selected loci could distinguish between the normal and the sick well in each cancer, and the results from the independent test sets demonstrated that the classification model constructed by loci from HyDML had better generalization ability.

Additionally, comprehensive investigation of the pDML showed that different cancers shared some common patterns in methylation variability at CpG locus resolution and revealed the potential similarities in different cancers. We found that same tissues share more abnormal methylation patterns with different subtypes of tumorigenesis, such as KIRC and KIRP, and LUAD and LUSC. This may indicate that the tissue specificity of methylation is preserved even when the tissue is cancerous. We also found a locus (cg02829688), which was hypermethylated in 13 cancers, located in a functional region on the genome, and could be of great potential to be an oncogenesis biomarker. Enriched motifs analysis from the background sequences of pDML revealed the potential influence on transcription function by CpG methylation, and the most significantly enriched motif, IRF3, has been reported playing a vital role in tumorigenesis. Through pathway analysis, some pan-cancer–related pathways were also determined, which have been reported playing a vital role in start, development, and metastasis of tumors.

As an import epigenetic mark, DNA methylation has been widely investigated to deepen our understanding of its mechanism and correlation with human illness, and it is possible to analyze methylation at all levels with the massive data generated by high-throughput detection technology. However, how to effectively identify DML from high-throughput methylation data is still a tough challenge even if feature selection methods have been extensively explored in the context of gene expression data. Innovatively, combining the instance perturbation and function diversity, the newly proposed method HyDML achieved effective identification of DML, and this demonstrated that ensemble feature selection could be used in dimension reduction for large-scale biological data. This will not only facilitate future early diagnosis of cancers based on the DNA methylation signatures but also enable additional investigations into the utilization of feature selection on other biomarker analysis domains. In the future, we will continue to study in depth the application of machine learning in biomarker identification and achieve better selection and prediction effect by combining more related information.

## Conclusion

In this article, a hybrid ensemble approach is proposed by incorporating instance perturbation and multiple functions to identify differential methylation sites across 13 cancers from TCGA. The specially designed framework makes it possible to select robust differential methylation sites, which not only improves the accuracy of the classifier built by the selected sites, but also enhances the confidence of domain experts to implement biological validations. Further intensive analysis reveals that different cancer types have common methylation patterns, and part of the differential methylation sites shared in pan-cancers is of great potential to be crucial in the early diagnosis of cancers. All findings demonstrate that abnormal DNA methylation could be regarded as a marker that expresses the difference between the normal and the sick.

## Data Availability

The data sets and materials for this study can be found in the following links:

HM 450 methylation data: https://tcga-data.nci.nih.gov/tcga/

Independent test sets:

1. For BRCA: https://www.ncbi.nlm.nih.gov/geo/query/acc.cgi?acc=GSE52635
2. For LIHC: https://www.ncbi.nlm.nih.gov/geo/query/acc.cgi?acc= GSE54503
3. For LUAD: https://www.ncbi.nlm.nih.gov/geo/query/acc.cgi?acc=GSE66836

Source codes of HyDML, DML files, and single-nucleotide polymorphism files have been provided as an open source available at https://github.com/TQBio/HyDML.git.

## Author Contributions

QT, JZ, and SF conceived and designed the experiments. QT, ZY, YF, JT, YS, and SF performed the analysis and edited the manuscript. JZ and SF led the research and reviewed the manuscript. All authors read and approved the manuscript.

## Funding

This work was supported by the National Natural Science Foundation of China (no. 61503061 and no. 61872063) and the Fundamental Research Funds for the Central Universities (no. ZYGX2016J102).

## Conflict of Interest Statement

The authors declare that the research was conducted in the absence of any commercial or financial relationships that could be construed as a potential conflict of interest.

## Supplementary Material

Figure S1
**(A)** The distribution density of DML in 22 pairs of autosomal chromosomes in 13 cancers. **(B)** The distribution of DML in different functional regions in 13 cancers. **(C)** The distribution of DML in CpG island and non-CpG island in 13 cancers.

Figure S2 Unsupervised hierarchical clustering of mutual DML in 13 cancers using similarity measure with Jaccard distance.

Figure S3 The AUC changed when the number of selected loci gradually reduced in each cancer. All the results show that HyDML performed better than single-feature selection methods as it can select more robust loci for distinguish normal and tumor samples.

Figure S4 The t-SNE clustering results with the loci that were uniquely selected by the three methods, HyDML, FastDMA, and RnBeads. Each row represents the loci from the corresponding cancer type, and each column represents the result of corresponding method.
