# Supplementary figures and images for: A Hybrid Ensemble Approach for Identifying Robust Differentially Methylated Loci in Pan-Cancers
Source: Front Genet. 2019 Sep 5;10:774. doi: 10.3389/fgene.2019.00774 (PMC6739624; doi:10.3389/fgene.2019.00774)

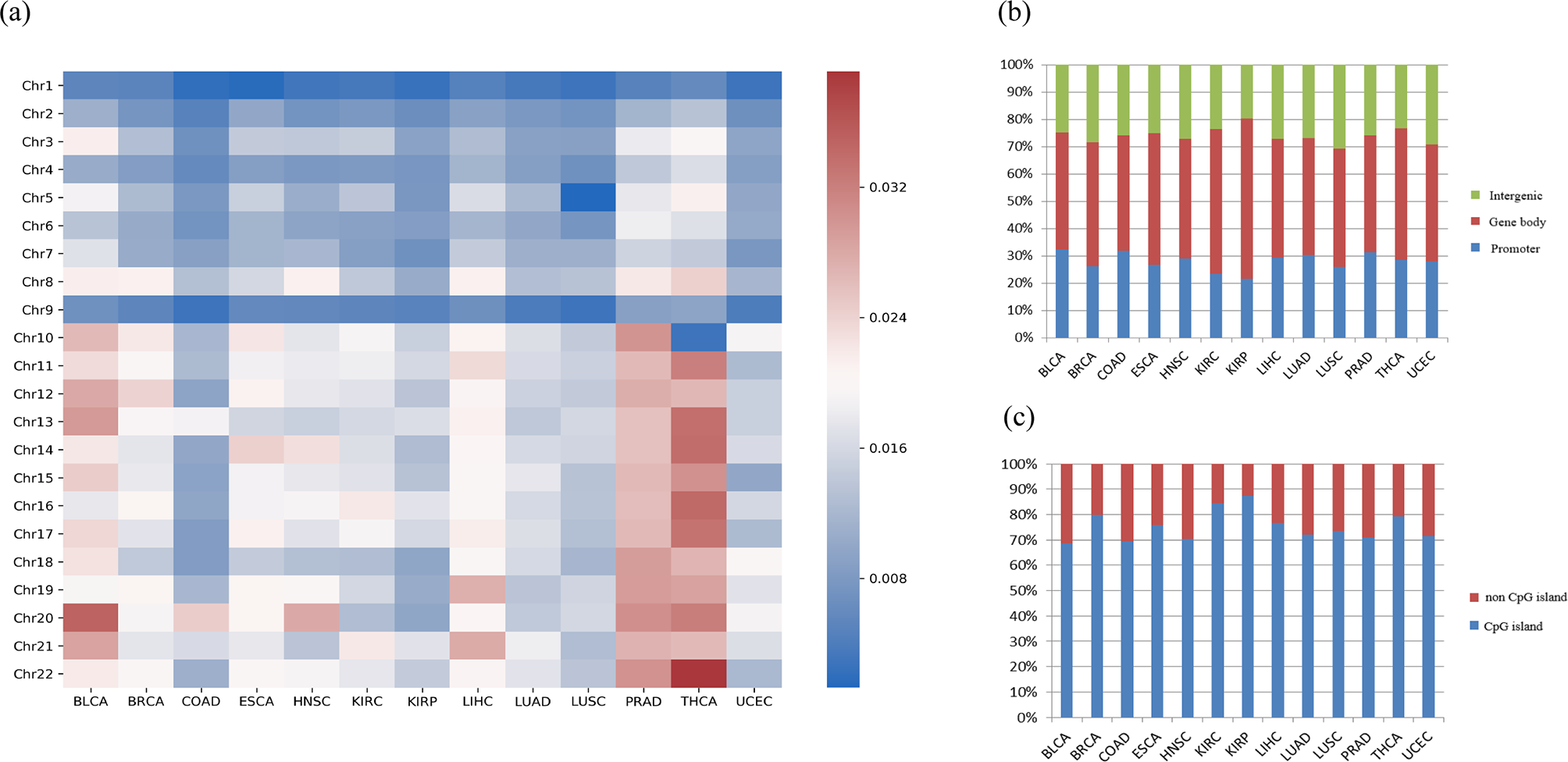

Supplement: Figure S1 — (A) The distribution density of DML in 22 pairs of autosomal chromosomes in 13 cancers. (B) The distribution of DML in different functional regions in 13 cancers. (C) The distribution of DML in CpG island and non-CpG island in 13 cancers. [file Image_1.tif]

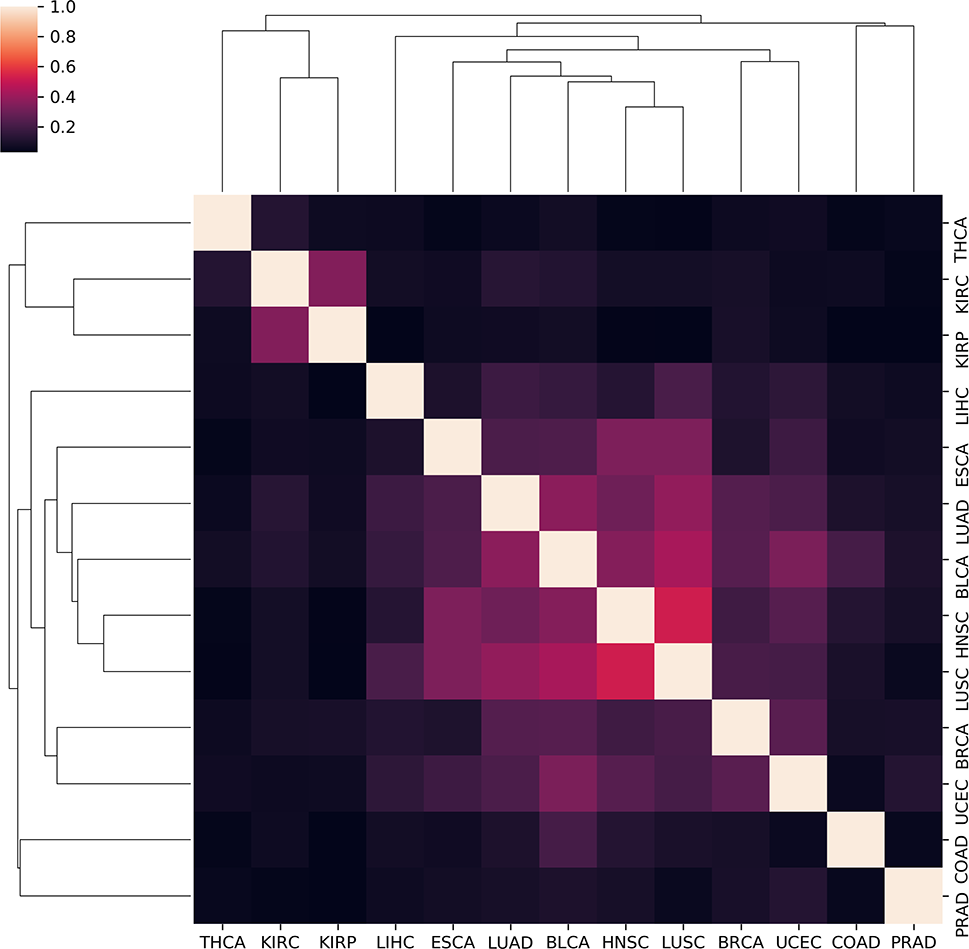

Supplement: Figure S2 — Unsupervised hierarchical clustering of mutual DML in 13 cancers using similarity measure with Jaccard distance. [file Image_2.tif]

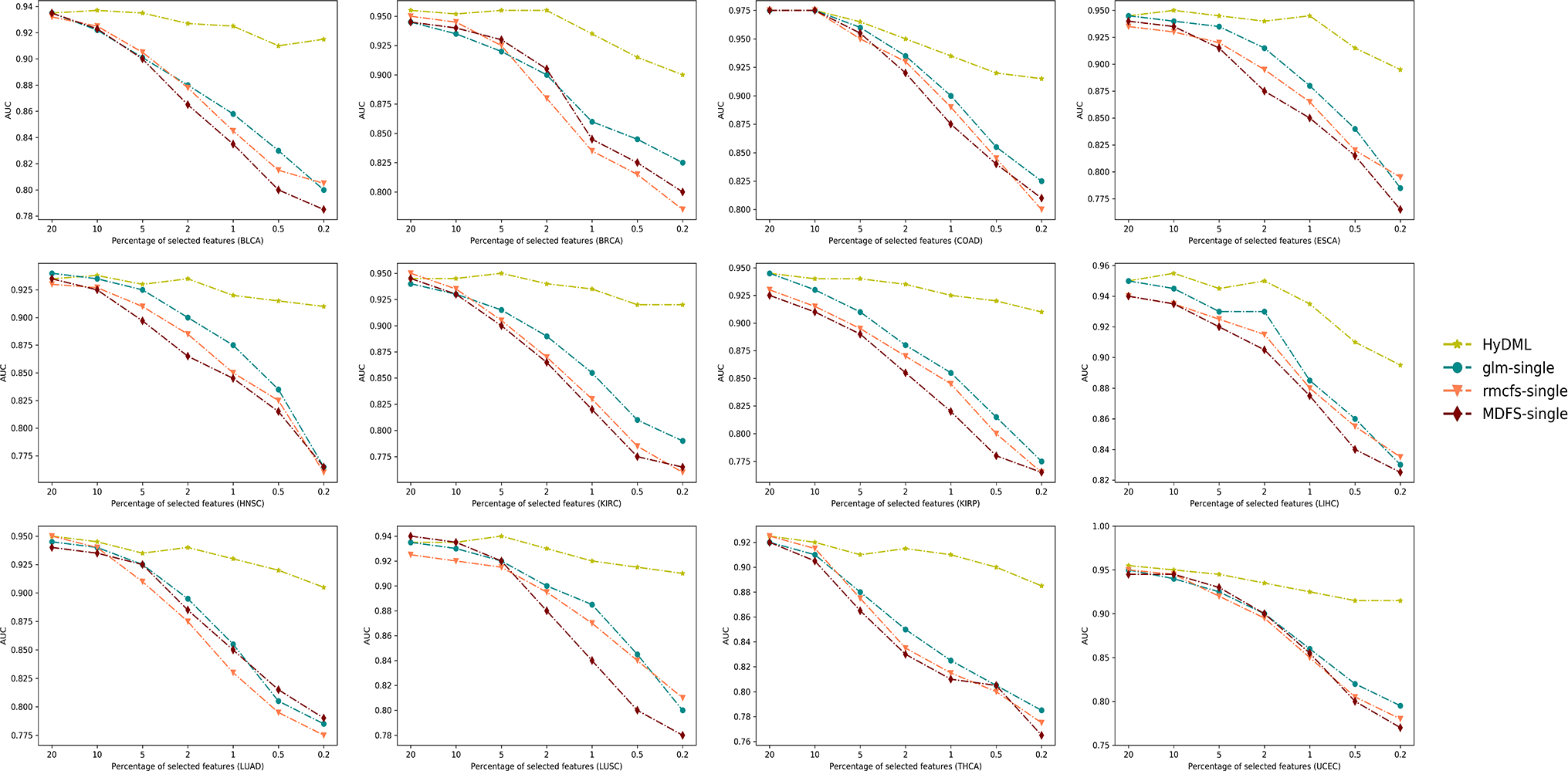

Supplement: Figure S3 — The AUC changed when the number of selected loci gradually reduced in each cancer. All the results show that HyDML performed better than single-feature selection methods as it can select more robust loci for distinguish normal and tumor samples. [file Image_3.tif]

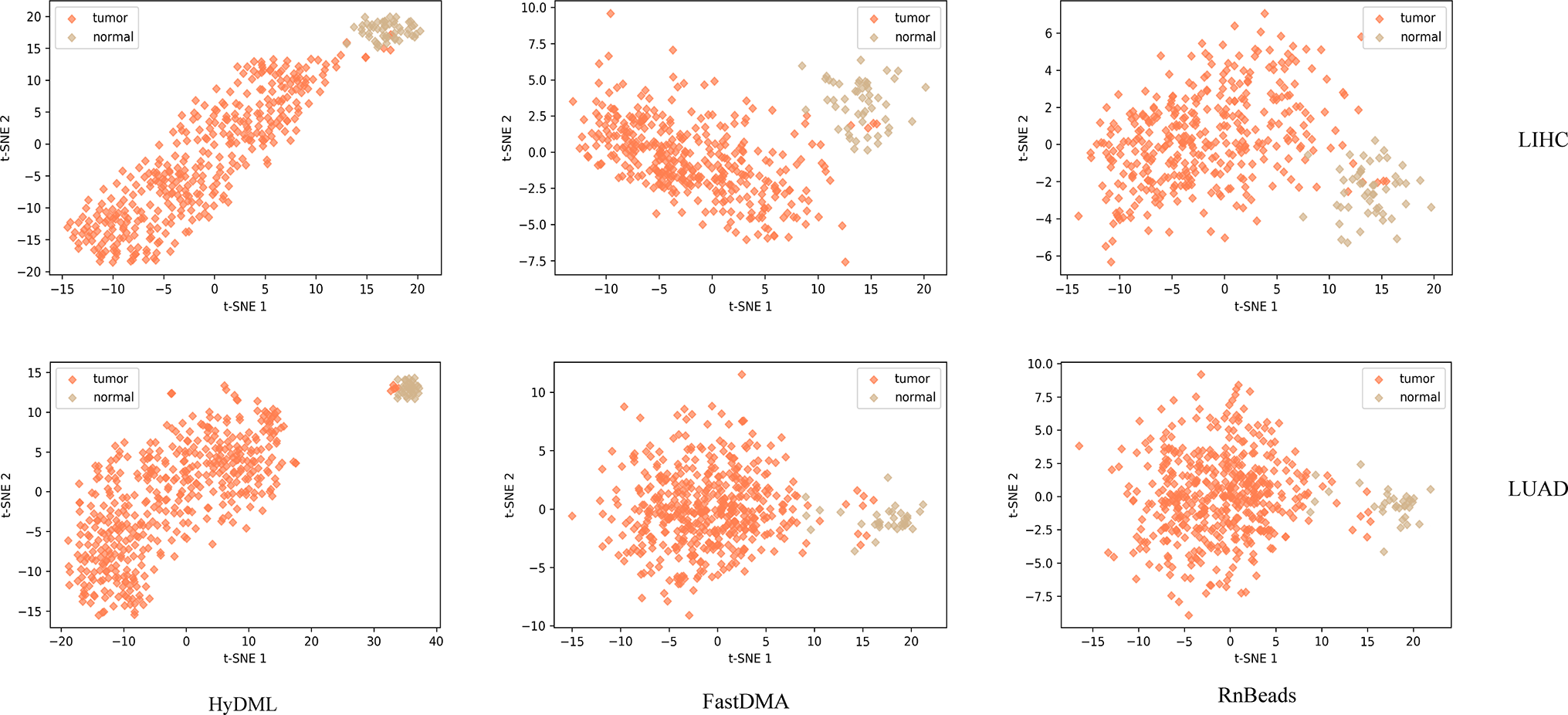

Supplement: Figure S4 — The t-SNE clustering results with the loci that were uniquely selected by the three methods, HyDML, FastDMA, and RnBeads. Each row represents the loci from the corresponding cancer type, and each column represents the result of corresponding method. [file Image_4.tif]
